# Genome and Ontogenetic-Based Transcriptomic Analyses of the Flesh Fly, *Sarcophaga bullata*

**DOI:** 10.1534/g3.119.400148

**Published:** 2019-03-29

**Authors:** Ellen O. Martinson, Justin Peyton, Yogeshwar D. Kelkar, Emily C. Jennings, Joshua B. Benoit, John H. Werren, David L. Denlinger

**Affiliations:** * Biology Department, University of Rochester, Rochester, NY 14627; † Departments of Evolution, Ecology and Organismal Biology and Entomology, Ohio State University, Columbus, OH 43210; ‡ Departments of Biological Sciences, University of Cincinnati, Cincinnati, OH 45221

**Keywords:** Diptera, *Sarcophaga bullata*, diapause, host-parasitoid interactions, ontogenesis, forensics, stress tolerance

## Abstract

The flesh fly, *Sarcophaga bullata*, is a widely-used model for examining the physiology of insect diapause, development, stress tolerance, neurobiology, and host-parasitoid interactions. Flies in this taxon are implicated in myiasis (larval infection of vertebrates) and feed on carrion, aspects that are important in forensic studies. Here we present the genome of *S. bullata*, along with developmental- and reproduction-based RNA-Seq analyses. We predict 15,768 protein coding genes, identify orthology in relation to closely related flies, and establish sex and developmental-specific gene sets based on our RNA-Seq analyses. Genomic sequences, predicted genes, and sequencing data sets have been deposited at the National Center for Biotechnology Information. Our results provide groundwork for genomic studies that will expand the flesh fly’s utility as a model system.

*Sarcophaga bullata* Parker (Diptera: Sarcophagidae), sometimes referred to as *Neobellieria bullata* (but see Stamper *et al.*, 2012), is a flesh fly widely distributed across North America (Byrd and Castner 2009). Like other members of this family, the female gives birth to active first instar larvae that are deposited on carrion and thus can begin to feed immediately. This life style exposes the larvae to a plethora of stresses including anoxia, temperature extremes, and pathogens that are caused by large numbers of feeding individuals within the small area of a single carcass. *S. bullata* and a sister species, *S. crassipalpis*, are easy to rear in the laboratory, making them useful models for diapause, cold tolerance, and other stress responses. The close association of flesh flies with garbage, rotting carcasses, and feces suggests these flies may serve as mechanical vectors of disease (Graczyk *et al.*, 2001), and the occasional deposition of larvae in vertebrate wounds (myiasis) rank these flies from a minor to significant species of medical and veterinary importance. Additionally, they serve as a key indicator species in forensic studies (Rivers and Dahlem 2014).

Substantial literature is devoted to understanding the fly’s diapause and regulation of seasonality (Denlinger 1972; Henrich and Denlinger 1982; Rinehart *et al.*, 2007; Spacht *et al.*, 2018; Reynolds *et al.*, 2017) seasonal acclimation (Adedokun and Denlinger 1984, Chen *et al.*, 1987), rapid cold hardening (Lee *et al.*, 1987; Chen *et al.*, 1987; Michaud and Denlinger 2006), and acute cold stress (Joplin *et al.* 1990; Chen *et al.*, 1990; Yocum *et al.*, 1994). *S. bullata* also offers a potent model for probing an interesting maternal effect. If a female *S. bullata* has experienced pupal diapause, none of her progeny are capable of entering diapause, even if reared in a strong diapause-inducing environment (Henrich and Denlinger, 1982, Rockey *et al.*, 1991). Mounting evidence suggests that epigenetic aspects act in the regulation of this maternal effect (Reynolds *et al.*, 2013; Reynolds *et al.*, 2016).

The fact that *S. bullata* is a favored host of the jewel wasp *Nasonia vitripennis* (Rivers and Denlinger 1995; Desjardins *et al.*, 2010; Werren and Loehlin 2009a) adds further experimental importance to *S. bullata* as a model species, as *Nasonia* is emerging as a genetic model for parasitoid genetics, evolution and development (Lynch 2015; Werren and Loehlin 2009b). Combining the sequencing of *S. bullata* with genomic information on *N. vitripennis* offers a powerful platform for studying host-parasitoid interactions (Danneels *et al.* 2013; Martinson *et al.*, 2014; Mrinalini *et al.*, 2015; Siebert *et al.*, 2015; Martinson and Werren 2018).

Previous transcriptomic and genomics studies have focused on carrion-feeding flies closely related to *S. bullata*. In *S. crassipalpis*, transcriptomes have been used to examine responses related to diapause and cold tolerance (Ragland *et al.*, 2010; Teets *et al.* 2012), and a transcriptome has recently been generated for *S. peregrina* (Kim *et al.* 2018). Beyond the genus *Sarcophaga* and the family Sarcophagidae, transcriptomic and genomic resources have been developed for other related carrion-feeding flies (Sze *et al.* 2012; Anstead *et al.* 2015; Wang *et al.* 2015). The most extensively studied is the blow fly, *Lucilia sericata* (Calliphoridae): which has an assembled genome and transcriptome data (Sze *et al.* 2012; Anstead *et al.* 2015; Anstead *et al.* 2016). Thus, a growing body of literature is emerging for probing the biology of these two closely-related families, the Sarcophagidae and Calliphoridae.

We anticipate that the sequenced and annotated genome of *S. bullata* presented here will enhance the research potential of this important model system. Combining raw reads that were reported previously in a study focusing on sex chromosomes in *S. bullata* and other Diptera (Vicoso and Bachtrog 2013) with new Illumina and PacBio sequencing, we provide a well assembled genome with sex- and developmental-specific RNA-Seq analyses to identify genes associated with reproductive biology and ontogenesis.

## Methods and Materials

### Source of Flies

All flies originated from a colony of *S. bullata* collected in Columbus, Ohio by the Denlinger laboratory at Ohio State University and subsequently maintained in culture by Carolina Biological Supply Co. (Burlington, NC). Samples prepared by the Bachtrog laboratory at the University of California, Berkeley, were purchased directly from Carolina Biological Supply and are hereafter referred to as the Carolina strain. Samples prepared by the Werren laboratory at the University of Rochester also were derived from flies purchased from Carolina Supply Company and maintained for several years in the Werren laboratory prior to use in this study and are hereafter referred to as the Werren strain. While not highly inbred, both strains originated from the same source colony that was sourced from a small group of wild caught individuals limiting the variation between strains.

### Illumina and PacBio Sequencing

DNA from a single male and a single female of the Carolina strain were extracted in the Bachtrog laboratory for another study, details of which can be found in Vicoso and Bachtrog (2015). A second collection of DNA from three pooled females of the Werren strain was extracted with DNeasy Blood and Tissue kit in the Werren laboratory. Illumina library preparation and sequencing were completed at University of Rochester Genomics Research Center and resulted in 21 billion raw bases in paired-end, 100bp reads from the Werren strain.

For PacBio sequencing, DNA was extracted from the Werren strain using a Gentra Puregene Tissue kit (Qiagen) with a modified protocol in the Denlinger lab. In short, our protocol differed from the manufacturer’s protocol in the following ways: all references to vortexing in the original protocol were replaced with mixing by tube inversions; Proteinase-K incubation was performed with continual inversion at 55° on a Max Rotator setting 5 (Lab Line, Mumbai, India); DNA was precipitated using several small aliquots of isopropanol chilled to -20° instead of a single aliquot of room-temperature isopropanol. An aliquot of DNA was run on a 0.1% agarose gel to check for degradation. DNA was quantified utilizing a Qubit 2.0 Fluorometer and double stranded DNA high sensitivity kit (Life Technologies Grand Island, NY) following the manufacturer’s instructions. Samples were sent to the Duke Genome Sequencing and Analysis Core Resource (Durham, NC) for library preparation and sequencing of fifty SMRT cells.

### Quality Control

PacBio reads were split into sub-reads and filtered for quality score at the Duke facility. Sub-reads were further filtered to remove low quality sequences. FastQC was used throughout to monitor quality control of Illumina reads. FastQC identified two issues affecting quality of the raw reads. First, there was an unexpected A/T/G/C distribution in the first few 5′ bases of reads obtained from the Carolina strain; this was addressed by trimming off these bases. Second, genomic reads obtained from the Werren strain had an unexpectedly high concentration of certain k-mers at particular places along the read; each read containing these k-mers was trimmed from the 5′ end. Reads were further filtered for primer contamination, quality (minimum Phred score of 28), and size (minimum 75bp) with Trimmomatic (Bolger *et al.*, 2014).

Different k-mer filtering approaches were tried with the k-mer filtering program supplied with SOAPdenovo and were assessed based on the quality of the assembly produced (see Genome Assembly). K-mers occurring at lower than expected frequency are often the result of sequencing errors. It is then possible to correct, trim, or filter reads containing these rare k-mers.. The assembly that was chosen was created from the reads filtered by counting the k-mers of length 19 produced from the Werren and Carolina reads.

### Genome Assembly

Utilizing reads from three filtering approaches, three assemblers, and different settings resulted in the creation of over 250 assemblies. Many were discarded based on size and continuity of the assembly. Reapr (Hunt *et al.*, 2013) was used to examine differences among the retained assemblies and to break possible miss-assemblies. One short read assembly was chosen based on the number of “error free bases” as reported by Reapr. This final assembly was produced by the assembler SOAPdenovo2 (Luo *et al.*, 2012) with the following settings: [-d 1 -k 65] during sparse pregraph phase, [-R] during contig phase, [-k 27] during map phase, and [-F -V] during the scaff phase. The chosen assembly was filtered for vector contamination using Vecscreen. The Illumina-based assembly was then improved with PacBio reads using PBJelly (English *et al.*, 2012) with default settings. Scaffolds were assigned to specific chromosomes-based on those previously identified by Vicoso and Bachtrog (2015).

### Annotation

Gene annotation was accomplished using the MAKER annotation pipeline (Cantarel *et al.*, 2008) to map protein homology data, expressed sequence tag evidence and *ab initio* gene predictions to the draft genome. Protein homology data were provided by Swiss-prot (UniProt Consortium 2015). To avoid spurious matches to repetitive regions of the genome, RepeatMasker was used to mask low-complexity regions (Smit *et al.*, 1996-2010). In addition to the included libraries, a custom repeat library for use with RepeatMasker was created with RepeatModeler (Smit and Hubley 2008-2015), RECON (Bao and Eddy 2002), RepeatScout (Smit and Hubley, 2008-2015), and TRF (Smit and Hubley, 2008-2010). Filtered RNA-Seq reads were mapped to the genome with Bowtie2 (Langmead and Salzberg 2012), junctions were mapped with TopHat (Trapnell *et al.*, 2009), and putative transcripts were assembled with Cufflinks (Trapnell *et al.*, 2010). The output from TopHat and Cufflinks were converted into gff files and passed to Maker as expressed sequence tag evidence. An iterative approach with three rounds of training was used with MAKER and the training of the *ab initio* predictors SNAP (Korf 2004) and AUGUSTUS (Stanke *et al.*, 2006). For the first round, SNAP was not used and the included ‘fly’ hidden Markov model was used in AUGUSTUS. In subsequent rounds, gene models predicted in the previous round of MAKER were used to generate hidden Markov models for SNAP and AUGUSTUS.

Of the 14,375 gene models that were identified, 2,717 were found to be composed of multiple smaller gene models, in that they contained transcripts with completely non-overlapping coordinates, often on opposing strands. These ‘concatenated’ gene models were broken into the smaller ‘constituent’ gene models. We sought to verify the validity of fragmenting the ‘concatenated’ gene models when the resulting ‘constituent’ gene models were found to be on the same strand. We identified hymenopteran proteins in NCBI’s nr database that showed sequence similarity to these ‘constituent’ gene models using BLASTp with a e-value cut-off of e10^−9^ and > 80% sequence similarity and >80% coverage to the ‘constituent’ gene models. For all the ‘constituent’ gene models for which homologs were present in other hymenopteran genomes, we identified hymenopteran proteins that were of similar length (± 20%). Consequently, all concatenated gene models were broken into their constituent gene models.

Gene models were further filtered to remove those with internal stop codons and very short (<10 bp) coding exons, resulting in a final set of 15,763 gene models. Quality of the genome and predicted gene models were assessed by examining the presence of Benchmarking Universal Single-Copy Orthologs (BUSCO) developed for Diptera (Simão *et al.*, 2015). CEGMA-based analyses were also utilized to examine for completeness of the genome (Parra *et al.*, 2008). Functional annotation was accomplished with Blast2GO (Conesa *et al.*, 2005; Conesa and Gotz 2008) using the *S. bullata* gene models and the top 50 BLAST hits (BLASTx, e-value < 10^−5^) from the NCBI nr database.

### Comparative Genomics

A species phylogeny was reconstructed to determine evolutionary relationships among eight dipteran species. The official protein set of *Lucilia cuprina* (NCBI, GCF_000699065.1), *Musca domestica* (NCBI, GCF_000371365.1), *Glossina morsitans* (VectorBase, GmorY1.8), *Drosophila melanogaster* (NCBI, GCF_000001215.4), *Mayetiola destructor* (i5k, Mdes_1.0), *Aedes aegypti* (VectorBase, AaegL3.3), and *Anopheles gambiae* (VectorBase, AgamP3) were downloaded from NCBI, VectorBase, or i5k and searched against the *S. bullata* gene set using BLASTp. A significant e-value cut-off ≤1e^-5^ was applied and only genes that had a single hit across all eight species were included in further analysis. A total of 343 individual proteins (161,034 amino acids, Table S1) met these criteria and were aligned with MAFFT (Katoh *et al.*, 2002) using default settings, and alignments were trimmed using gBlocks to remove gaps (Talavera and Castresana 2007). The aligned single-copy protein-coding genes were then concatenated and the phylogeny was reconstructed using RAxML version 8.2.8 (Stamatakis 2006) with the PROTGAMMAWAG model and 100 bootstrap replicates. The phylogeny was visualized with FigTree version 1.4.2 (http://tree.bio.ed.ac.uk/software/figtree/). Orthologous groups of genes were also determined among the eight species using OrthoFinder (v 2.2.7) (Emms and Kelly 2015) using default settings.

### Expression Analysis

To determine sex- and development-specific gene sets, gene expression of the entire gene set was calculated for testes, ovaries, adult female, adult male, adult male carcass (adult males with testes removed), adult female carcass (adult females with ovaries removed), larva, and pupa at three and six days after pupariation. The three- and six-day pupal samples were originally sequenced for a previous study (Martinson *et al.*, 2014). Samples were collected and RNA isolated for the testes, ovaries, adult female, adult male, adult male carcass, adult female carcass in the Bachtrog laboratory for a previous study, details of which can be found in Vicoso and Bachtrog (2013). RNA from the larva and second ovary sample were collected in the Werren Lab. Total RNA was extracted using TRIzol Reagent (Ambion) per manufacturer’s protocol, followed by quantification and quality checking using Agilent 2100 Bioanalyzer. TruSeq mRNA (Illumina) library construction and 100bp paired-end sequencing on Illumina HiSeq 2500 platform were performed by University of Rochester Genomics Research Center (URGRC). cDNA for each sample was indexed with a unique adapter. Each library was normalized by equimolar multiplexing before sequencing at ∼1 library/10^th^ of a lane.

RNA reads were mapped to gene models using the Burrows-Wheeler Aligner (BWA v.0.7.8), allowing for two mismatches per raw read (-n 2) (Li and Durbin 2009). Cufflinks v.2.2.0 was used to calculate FPKM (Fragments Per Kilobase of transcript per Million mapped reads) for each gene (Trapnell *et al.*, 2010) and count data were calculated using HTSeq (v. 0.9.0) (Anders *et al.* 2015). A single replicate of each library was sequenced, with exception of pupa, for the primary purpose of genome annotation, however we report some differential expression analyses here to provide some preliminary results for future studies. Genes were considered differentially expressed if there was a fourfold change in expression with at least one library in the comparison having a minimum of 10 FPKM. Genes were considered specific to a life stage if they had an expression of >50 FPKM and were significantly upregulated against all other life stages. GO enrichment analyses were performed using BiNGO in Cytoscape with an adjusted p-value < 0.01 (Maere *et al.*, 2005).

### Data availability

All raw sequencing data from this project is available as part of the flesh fly genome project (NCBI Bioproject: PRJNA476317), with the exception of the RNA-Seq data for three- and six-day pupa, which are available in BioProject PRJNA255811. Tables containing the annotations, GO terms, counts, and FPKM values for each predicted genes as well as lists of differentially expressed genes and overrepresented GO terms are available on Figshare. Supplemental material available at Figshare: https://doi.org/10.25387/g3.7798637.

## Results and Discussion

### Sequencing and Assembly

Illumina sequencing resulted in 428 million reads totaling 40 billion bases (67X coverage). About 83% of the reads were retained after quality control and filtering (Table 1). PacBio sequencing yielded 20 million size-selected reads totaling 14 billion bases (23X coverage).

**Table 1:** Summary of Illumina read filtering

	Reads (#)	Bases (#)	Reads (%)	Bases (%)
Raw	428,355,968	40,615,616,800	100	100
Quality Filtered	414,083,892	37,890,395,975	96.67	93.29
k-mer Filtered	376,681,172	33,610,109,059	87.94	82.75

Different assembly protocols resulted in short read assemblies ranging in size from 230 million bases to 3.8 billion bases, *i.e.*, 39–640% of the 593 million bases measured by flow cytometry (Pimsler *et al.*, 2014), divided among 2n = 12 chromosomes (Bultmann and Mezzanotte 1987). Reapr was used to examine the quality of assembly and trim the short-read assemblies at sites of likely misassembly, thus resulting in substantive changes to the chosen assembly. Trimming reduced the number of non-gap bases from 439Mbp to 427Mbp. Reapr broke many scaffolds, increasing their number from 328K to 345K and reducing the N50 from 11.5Kbp to 9.3Kbp. PacBio reads were used with PBJelly to fill gaps, extend contigs and further scaffold the contigs. PBJelly provided substantial improvements in size and continuity. The number of non-gap bases increased from 427Mbp to 519Mbp and the N50 increased from 9.3Kbp to 29.5Kbp.

To help reduce sequencing contamination, scaffolds were screened against the UniVec database maintained by NCBI. Sequences (n = 117) that had suspected contamination were either trimmed or removed altogether. Lastly, genomic sequences were examined for potential microbial contamination based on methods developed for other invertebrate genomes (Benoit *et al.*, 2016; Poynton *et al.*, 2018). The final assembly, which consisted of 42,093 scaffolds (522 Mbp), was used for gene prediction and to establish the draft genome for *S. bullata*. This is 12% smaller than the predicted genome (593 Mbp, Pimsler *et al.*, 2014), a common result for first assembly of genomes (Anstead *et al.*, 2016; Benoit *et al.*, 2016; Attardo *et al.*, 2014).

**Figure 1:**
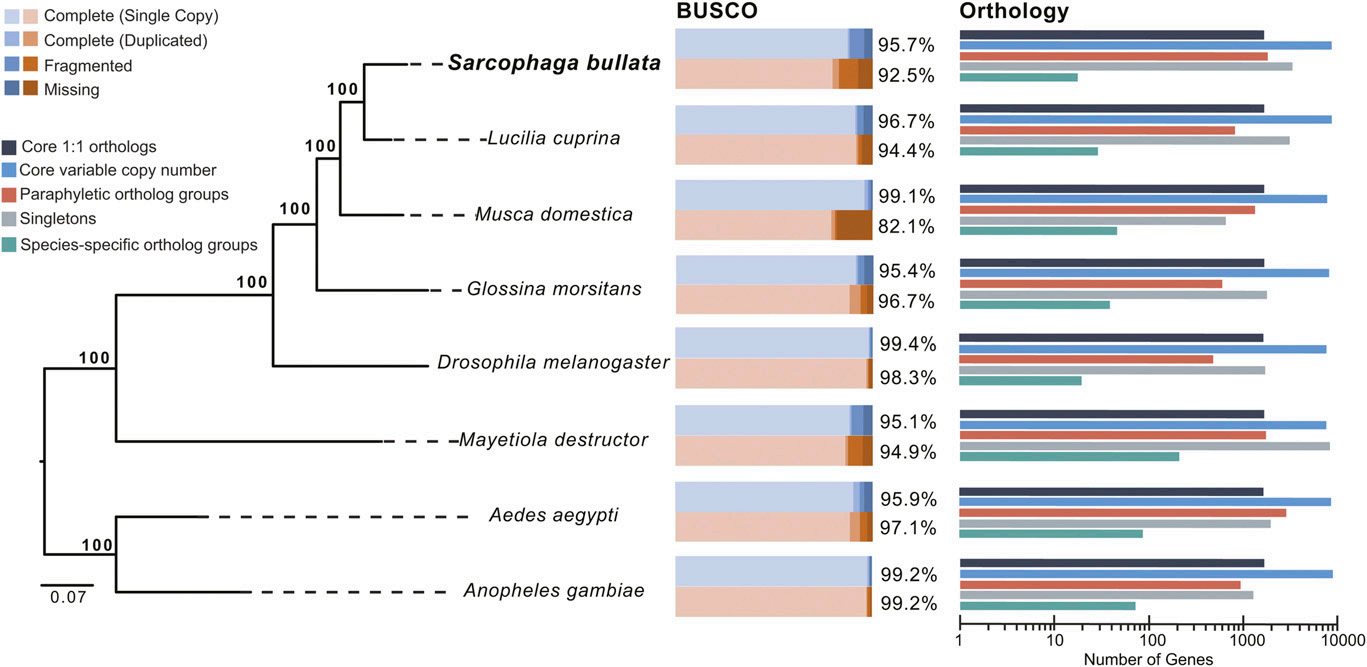
Phylogenetic placement and genomic comparisons for *Sarcophaga bullata* and other fly species. Left, The phylogenetic analysis places *S. bullata* as a sister species to the bottle fly, *Lucilia cuprina*. The phylogeny is built using RAxML and it is based on amino acid sequences from 343 single-copy genes that are present in all eight species. Bootstrap values are shown for every node. Middle, Benchmarking Universal Single-Copy Orthologs (BUSCO, Simão *et al.* 2015) analyses based on the dipteran dataset (odb8). The dataset was searched against the entire genome sequence (blue) and the annotated gene set (red). Right, Orthology-based analyses of protein coding genes between eight fly species determined the number of genes in single-copy core (*i.e.*, found in all species evaluated) clusters, variable-copy number core clusters, paraphyletic clusters (non-core, non-species specific), singleton, and species-specific clusters, based on OrthoFinder (Emms and Kelly, 2015).

To assess quality of the assembly, two different programs were used to scan for orthologs common to eukaryotes. Both found a high percentage of orthologs, thus indicating a well-assembled genome including most of the protein coding regions. BUSCO found just under 96% of the searched orthologs (Figure 1), a result similar to many of the published dipteran genomes (Anstead *et al.*, 2015; Attardo *et al.*, 2014; Scott *et al.*, 2014). There was evidence for 4% duplication, a relatively small percentage given the challenge of assembling multiple individuals into one assembly. CEGMA reported finding partial matches for >98% of the orthologs and full length matches for 92% of the orthologs. Thus, the quality of the genome is sufficient for subsequent analyses. Scaffolds that matched previously identified chromosomes are shown in Table S2. Approximately 15% of the scaffolds can be directly assigned to chromosomes based on those previously identified (Vicoso and Bachtrog 2015) and 56% show partial matches with less confidence to specific chromosomes. RNA-Seq reads mapped to these scaffolds that have been directly assigned revealed enriched expression for the male-associated library in unassigned chromosome and Chromosome 4.

### Annotation

To avoid spurious matches to repetitive regions, RepeatMasker was used to soft mask repetitive regions of the genome. The *D. melanogaster* RepeatMasker library (Dm6) failed to mask retroelements or DNA transposons. A custom library masked 230K retroelements and 127K DNA transposons. This included interspersed repeats, simple repeats, and other types of repetitive regions; a total of 148Mbp (31%) were masked (Table 2). These results are similar to *D. melanogaster* (29%) (http://www.repeatmasker.org/) and *Lucilia cuprina* (33%) (Anstead *et al.*, 2015) and lower than reported for *Musca domestica* (52%) (Scott *et al.*, 2014).

**Table 2:** Summary of different types of repeat elements

Type	Number	Bases	% of Genome
SINEs	33,478	7,221,604	1.52
LINEs	179,696	32,921,812	6.91
LTR	17,235	6,433,260	1.35
DNA	127,299	19,969,616	4.19
Unclassified	397,153	59,304,762	12.45
Total interspersed		125,851,054	26.42
Small RNA	323	44,904	0.01
Simple repeats	416,660	18,229,578	3.83
Low complexity	89,350	4,553,468	0.96
Total		148,389,050	31.15

We predict 15,768 protein-coding genes. This is slightly higher than the 13,919 protein coding genes found in *D. melanogaster* (dos Santos *et al.*, 2015), 14,180 protein coding genes predicted in *M. domestica* (Scott *et al.*, 2014), and 12,445 in *G. morsitans* (Attardo *et al.*, 2014). The number of exons per gene is similar between *S. bullata* (4.6) and *M. domestica* (4.4). However, the introns in *M. domestica* are on average roughly twice as long as in *S. bullata* (Scott *et al.*, 2014). Intron length in *S. bullata* is closer to that of *D. melanogaster* and *G. morsitans* (Table 3). Differences in intron length and proportion of repeat elements may reflect differences in genome sizes: the genome of *M. domestica* is approximately twice the size of *S. bullata* and *G. morsitans* (Table 3).

**Table 3:** Comparison of exon and intron content of *Sarcophaga bullata* to other flies. Source: Dm: dos Santos *et al.*, 2015; Gm: International *Glossina* Genome Initiative 2014; Md: Scott *et al.*, 2014

Species	Genome size (Mb)	Exon Number	Exon Length (bp)	Intron Number	Intron Length (bp)
*Drosophila melanogaster*	200	77,682	539	58,537	1,700
*Glossina morsitans*	590	63,000	475	52,000	1,600
*Musca domestica*	1021	67,886	431	52,875	3,889
*Sarcophaga bullata*	593	66,485	422	52,110	1,989

### Gene Expression Analyses

**Figure 2:**
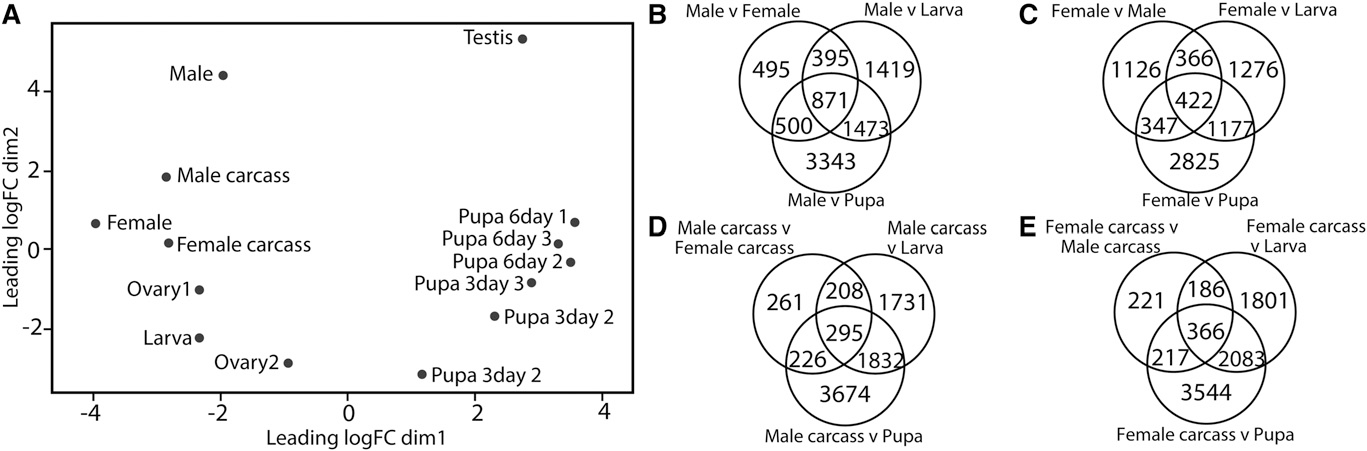
Gene expression analysis to determine sex- and developmental-specific gene sets in *S. bullata*. A) Multidimensional scaling analysis (MDS) showing clustering of sex and developmental stages. The MDS was performed on count data in EdgeR (Robinson *et al.* 2010). B-E) Venn diagrams categorizing differentially expressed genes in sex-specific gene sets displaying B) male-specific gene expression, C) female-specific gene expression, D) male carcass-specific gene expression, and E) female carcass-specific gene expression.

Of the 15,768 genes models in the *S. bullata* genome, 14,933 have >2 FPKM expression in at least one developmental stage and 12,310 were assigned at least one GO term (Table S3) When the gene expression profiles were mapped onto multidimensional scaling analysis (MDS), the testis had the most distinct gene expression profile (this is also reflected in the greater distance between gene expression profiles of male and male-carcass than between female and female-carcass) (Figure 2A), and is consistent with the fast evolution and differential expression previously shown in the testis of *D. melanogaster* (*e.g.*, Chintapalli *et al.*, 2007; Meiklejohn *et al.*, 2003). The expression profile for larva falls between the two ovary profiles, possibly because *S. bullata* is larviparous; larvae hatch within the uterus of the female, and she thus gives birth to active first instar larvae, rather than eggs. Thus, it is possible that one set of dissections of the female reproductive organs may have included some larvae, whereas the other may have contained only developing eggs (Figure 2A).

There were 871 genes differentially expressed in the male compared to female, larva, and 3-day pupa (as defined by fourfold change in expression) (Figure 2B). From this set we determined that 386 of these genes were male specific (as defined by significantly up-regulated genes with expression of >50 FPKM) (Table S4). Male-specific genes included four overrepresented GO terms, including sperm motility, cellular glucose homeostasis, and carbohydrate phosphorylation (Table S5). Only a third as many differentially expressed genes (295 genes) were present when the male-carcass (testes removed) was compared to the female-carcass (ovaries removed), larva, and 3-day pupa (Figure 2D and Table S4). This suggests that many of the differentially expressed genes in males were testis specific while the remaining are likely associated with other process such as generation of the seminal fluid by the accessory gland (Scolari *et al.* 2016) There were 52 overrepresented GO terms among the 161 male-carcass specific genes (Table S5). They included several metabolism categories including glycosaminoglycan catabolic process, fatty acid biosynthetic process, and regulation of synaptic transmission, results that are similar to what is seen in males of *Glossina* (Scolari *et al.* 2016). Male-carcass specific genes also contained several GO terms related to immune response such as defense response, peptidoglycan catabolic process, immune system process, and defense response to bacterium. These results may suggest that the sequenced individual had a pathogen triggering its immune response, or it could be similar to *D. melanogaster*, where the immune response in females is suppressed after mating (Short and Lazzaro 2010). The immune suppression could possibly last longer in larviparous *S. bullata* females, as an immune response could cause harm to hatched larvae within the female’s reproductive tract.

Females had a much smaller drop in the number of differentially expressed genes between the whole female (422 genes) and the female-carcass (366 genes), than observed between male and male-carcass (Figure 2C&E and Table S4). As expected, the 47 overrepresented GO terms among the 308 female-specific genes primarily deal with reproduction (*i.e.*, sexual reproduction, egg activation, and oogenesis), however several GO terms are also involved with cellular response to gamma radiation and DNA damage response (Table S5). The 20 overrepresented GO terms for the 139 female-carcass specific genes primarily focus on regulation of transport (regulation of transmembrane transport, regulation of ion transport, and positive regulation of transporter activity) and coagulation (hemolymph coagulation, coagulation, and hemostasis) (Table S5).

Among the 3,443 genes that did not have an ortholog outside of *S. bullata* (categorized as singletons or assigned to a *S. bullata* specific ortholog group), 930 (27%) were significantly sex biased in expression. However, proportions of female- and male-biased genes were relatively equal, with 483 significantly upregulated in males (85 genes), male carcass (40 genes) or testis (358 genes) and 447 upregulated in females (97 genes), female carcass (55 genes), or ovaries (295 genes).

### Conclusions

This study describes the assembly and annotation of the genome for *Sarcophaga bullata*, a key model organism in physiological and ecological studies that range from those on diapause to host-parasitoid interactions. The assembly of 522Mbp represents approximately 88% of the estimated 593 million bases measured by flow cytometry (Pimsler *et al.*, 2014). We predicted and analyzed 15,768 protein-coding genes that offer insights into the development and evolution of *S. bullata*. This was followed by sex- and development-specific RNA-Seq analyses that elucidate aspects underlying reproduction and ontogenic progression. Genomic scaffolds were assigned to specific chromosomes by comparison to those identified in previous studies (Vicoso and Bachtrog 2015). These combined genomic and RNA-Seq resources offer a platform to enhance future studies of *S. bullata* in endocrinology, stress tolerance, diapause, diapause epigenetics, maternal effects, parasitoid-host biology, and could enhance development of new and improved tools for forensic studies.
